# A Study on the Psychological Wound of COVID-19 in University Students

**DOI:** 10.3389/fpsyg.2021.589927

**Published:** 2021-01-26

**Authors:** Isabel Padrón, Isabel Fraga, Lucía Vieitez, Carlos Montes, Estrella Romero

**Affiliations:** ^1^Cognitive Processes & Behavior Research Group, Department of Social Psychology, Basic Psychology, and Methodology, University of Santiago de Compostela, Santiago de Compostela, Spain; ^2^Department of Social, Psychology, Basic Psychology, and Methodology, University of Santiago de Compostela, Santiago de Compostela, Spain; ^3^UNDERISK, Department of Clinical Psychology and Psychobiology, University of Santiago de Compostela, Santiago de Compostela, Spain

**Keywords:** university students, psychological impact, COVID-19, stressors, coping, COnVIDa-20

## Abstract

An increasing number of studies have addressed the psychological impact of the COVID-19 crisis on the general population. Nevertheless, far less is known about the impact on specific populations such as university students, whose psychological vulnerability has been shown in previous research. This study sought to examine different indicators of mental health in university students during the Spanish lockdown; we also analyzed the main sources of stress perceived by students in relation to the COVID-19 crisis, and the coping strategies adopted when faced with the situation. Data was collected from 932 students (704 women) through a web-based platform. Measures of anxiety (i.e., GAD-7), depression (PHQ-9), irritability, and self-perceived change in mental health were administered, as well as ad hoc measures of stressors and coping strategies. Results indicated that students experienced considerable psychological problems during the confinement, with higher rates of emotional difficulties in women and undergraduate students than in men and postgraduates, respectively. Psychological distress was mainly related to several specific domains of stressors, as perceived by the participants: academic future, task overload, worsening of interpersonal conflicts, and restrictions in pleasant social contact; and far less related to the spread of the disease and its consequences for physical health. As regards coping strategies, both reframing skills and daily routines were shown to be the most effective. A path-analysis model integrating stressors, coping, and mental health revealed that coping strategies partially mediated the effect of stressors on psychological health. In general, results suggest that students’ psychological health was substantially affected by the COVID-19 situation and that the academic and relational changes were the most notable sources of stress. This study reinforces the need to monitor and promote mental health in university students to boost resilience in times of crisis. Our results on effective coping strategies may inform preventive programs aimed at helping students to deal with challenges like the COVID-19 pandemic.

## Introduction

In late December 2019, a new coronavirus disease (COVID-19) emerged in the Chinese city of Wuhan. This new disease, caused by SARS-CoV-2 (Severe Acute Respiratory Syndrome Coronavirus-2), spreads very easily from one person to another and thus rapidly affected other parts of China (Wang et al., 2020). Within a few weeks, the first cases emerged in other countries, and COVID-19 soon became a global threat. Indeed, in March 2020, the World Health Organization (WHO) declared COVID-19 to be a global pandemic. As of July 06, 2020 (the date of writing this article), about 9,843,073 confirmed cases, including 495,760 deaths, have been reported by the WHO (World Health Organization [WHO], 2020a). Following the initial outbreak in China, further outbreaks occurred in Italy and Spain, the first Western countries to be affected, and thus the first countries to face a problem that had hitherto seemed a very distant one to the populations of Western societies. In fact, at the time of writing, Spain is one of the three countries (after Belgium and the United Kingdom) with the highest rate of confirmed cases and deaths per million inhabitants (July 06, 2020, cf. World Health Organization [WHO], 2020c).

In response to the COVID-19 outbreak, many countries were forced to adopt severe restrictive measures to slow down its propagation. In the case of Spain, the Spanish government declared a state of emergency on March 14th, and a population lockdown became mandatory 2 days later, creating an unprecedented situation. Citizens only could leave their homes for food, to go to a pharmacy, and for other essential needs. Schools and universities were closed throughout the national territory. Employers (public and private) were obliged to work from home whenever possible, and many lost their jobs temporarily or permanently. In a matter of days, millions of people’s lives changed dramatically, leading to important questions about how the pandemic was affecting not only the physical but also the mental health of the population.

Research initially focused mainly on the impact of COVID-19 on physical health and its clinical characterization (e.g., Cao and Li, 2020; Lvov et al., 2020), with studies on the psychological impact of the quarantine subsequently beginning to appear (e.g., Cao et al., 2020; Wang and Zhao, 2020). Findings of all studies (the first of these on the Chinese population, and soon after on other populations of Western countries) revealed a significant and severe increase in depressive symptoms, anxiety, and stress levels due to confinement (e.g., Cao et al., 2020; Chen et al., 2020; Wang and Zhao, 2020) which was generally more pronounced in women than in men (Flesia et al., 2020; Rodríguez-Rey et al., 2020; Wang and Zhao, 2020). Moreover, as recent reviews suggest (Brooks et al., 2020; Hossain et al., 2020), other negative psychological effects, such as post-traumatic stress symptoms, anger, panic, irritability, low self-esteem, and lack of self-control, are commonly found among individuals affected by physical isolation. Data from other pandemics and natural disasters revealed similar effects (see Brooks et al., 2020, for a review). A recent technical report on the Spanish population between 18 and 75 years of age, starting 26 days after the first state of emergency, also revealed significant rates of symptoms of depression (22.1%) and anxiety (19.6%) (Valiente et al., 2020). Importantly, results from this and other studies (e.g., Flesia et al., 2020; Ozamiz-Etxebarria et al., 2020; Rodríguez-Rey et al., 2020) reveal that not all groups were affected in the same way, with young adults (18–24) being more affected compared to other age groups.

To design action plans aimed at protecting and helping citizens who may be affected in different ways by these types of situations, it is first necessary to establish the specific effects of the pandemic in different populations. Interestingly, although studies on the general population are accumulating, the impact on university students is still not well known. There is, however, an abundance of work indicating that most mental health disorders have first onset in young adulthood (e.g., Kessler et al., 2001; Eisenberg et al., 2007). Several studies have also reported that students have consistently higher levels of mental health problems than the general population (Overbeek et al., 2003; Zivin et al., 2009; Auerbach et al., 2016). Moreover, research in this field notes the importance of personal and psychosocial factors in the emergence and development of mental disorders in university students (Galindo et al., 2009). Besides academic issues, university students are exposed to multiple stressors which are unique to this developmental period, such as the abandonment of the family home, adjusting to new social and geographical environments, making new friends and social relationships, life-stage transitions, time management, economic resources, etc. (Beiter et al., 2015; Fried, 2020). The lockdown substantially affected these conditions, and the well-being of the university student population would also be expected to have been affected. In brief, university students constitute a population that is particularly vulnerable in terms of mental health, one that even before the pandemic showed a high prevalence of mental disorders. Moreover, many universities suspended normal class-based teaching and moved online, with the result that the lives of students changed drastically (Sahu, 2020) and their psychosocial functioning was negatively disrupted, thus altering the social integration of some individuals (Elmer et al., 2020). It is within this situation, then, that we ask how students’ psychological health might have been affected by the pandemic and the confinement.

Thus far only a few papers have addressed this issue specifically. Recent studies on Chinese undergraduate students reported higher levels of anxiety during the COVID-19 outbreak (e.g., Cao et al., 2020; Wang and Zhao, 2020). In the Cao et al. (2020) study, for example, the authors found that 24.9% of students experienced symptoms of anxiety, with 0.9% of cases being severe and 21.3% mild anxiety. Moreover, it was reported that some factors, such as place of residence, source of parental income, whether living with parents or not, and having a relative or an acquaintance infected with COVID-19, were associated with increased anxiety. Another recent study, looking at the initial psychological impact of the COVID-19 outbreak on a population of Spanish university students (77%), academic staff (13%), and administrative staff (9%), revealed significantly higher anxiety, depression, and stress scores in the students than in the other two groups (Odriozola-González et al., 2020). Meanwhile, Elmer et al. (2020) analyzed changes in social networks and mental health in a sample of Swiss undergraduate students during the COVID-19 crisis (2 weeks after the lockdown) in relation to the two preceding years. They also analyzed individual and social factors associated with changes in mental health during the outbreak. In line with previous work, students were found to feel more depressed, anxious, stressed, and lonely than half a year before, and these negative effects were more prominent in women. Also, concerns about family and friends, future careers, living alone, and having less social contact and support, were linked to worse mental health. Even though some studies have not found significant changes in students’ mental health during the crisis (Fried, 2020), the general results are congruent with previous research on the psychological impact of previous pandemics in China, in which students presented high levels of stress and anxiety during SARS (Jia et al., 2003; Wong et al., 2007) or N1H1 influenza (Li et al., 2011).

Although a small number of studies have focused on student populations, we know very little about the specific sources of stress that affected university students during the most acute stage of the crisis, and about the differential impact that these had on the psychological well-being of students. In addition to the stressors present in the general population, such as prolonged isolation periods, fear of infection, frustration and boredom, inadequate information (excess or confusing information), financial loss, and inadequate supplies, as noted by Brooks et al. (2020) and Hossain et al. (2020), university students were also greatly affected by significant academic changes resulting from the pandemic. All Spanish universities suspended face-to-face teaching and moved to online classes and examinations, a transition likely to have had a serious impact on students’ feelings of anxiety and uncertainty. As Sahu (2020) has observed, the quality of online education, and its consequences, is a critical issue here, because students without adequate Internet access will experience even more stress, which can have detrimental effects on their academic performance and achievement, as well as on their mental health. We might add that not all individuals possess equal levels of technological proficiency or confidence, and that the Internet itself was often slow and unreliable during this period, all of which meant that the interactive online environment was sometimes a less than optimal experience.

With respect to these specific sources of stress, a study by Okruszek et al. (2020) with Polish young adults found that the COVID-19 risk perception (e.g., contact risk, severe symptoms risk, and financial problem risk) and the feeling of loneliness led to affective responses to the situation during the first 2 weeks of the crisis. However, it is also interesting to note that some individual stressors, such as work overload, strict schedules, Fears of Missing Out on social life (FoMO) or competition among students might in fact have been mitigated during the crisis situation for some students (Elmer et al., 2020).

There is also little prior evidence about coping strategies used by young adults in this situation. Although the structure of coping strategies is still a controversial issue (Stanisławski, 2019), many different coping behaviors have been considered in the long tradition of research on stress: some of the coping behaviors are considered more “active,” i.e., directed to cognitively or behaviorally change the stressor (e.g., problem solving, cognitive reframing; Tobin et al., 1989; Gaudreau, 2017), while others are more “passive” or “disengaged” behaviors (e.g., distracting activities, substance use, social withdrawal). Apart from those well-known strategies, some other coping behaviors have been identified in collective crises; for example, following routines or involving in healthy habits (Fullana et al., 2020). Likewise, previous research has suggested the coping value of altruistic behaviors in wide-scale stressful situations (e.g., Sharma and Kar, 2019). Some decades ago, Midlarsky (1991) proposed that helping may be considered as a coping mechanism, which may be effective through different psychological processes; for example, orientation to others may distract the individual from own troubles; it may also enhance the sense of competence and self-efficacy and may provide a meaning to life in the middle of adversity; additionally, helping others may also promote social integration, and evoke reciprocal support from other people. Recent studies have also emphasized the coping function of helping (Vollhardt, 2009), and physiological and neurochemical pathways have been identified (e.g., activation of parasympathetic system, oxytocin levels, and dopaminergic activity) to explain the buffering effects of helping behavior (Raposa et al., 2016). During the COVID-19, some preliminary studies have also reported the use of helping as a coping mechanism (Balluerka et al., 2020). Nevertheless, research on coping during the pandemics is still limited; particularly, the impact of the various types of coping on students’ adjustment in the COVID-19 crisis is largely unknown.

For all the above reasons, the present study was conducted, and had three main goals. The first of these was to study the psychological impact of the COVID-19 pandemic on mental health in a sample of Spanish university students after 6 weeks of quarantine. The second was to analyze the main sources of stress associated with the COVID-19 context, including stressors arising from the pandemic, measures of social isolation, and changes experienced by students in the academic environment. Finally, we sought to address the issue of which coping strategies were used by students, and how such strategies were related to psychological health during the lockdown. Results were expected to help explore questions of how psychological health was affected during the acute part of the crisis, as well as which stressors and coping behaviors may explain the differences in mental adjustment when faced with the challenges of COVID-19.

## Materials and Methods

### Participants

This study forms part of the wider COnVIDa-20 project, which aims to identify the psychosocial needs and challenges, plus the skills and resources, of Spanish university students during the COVID-19 pandemic; this is the first report on the data from the COnVIDa-20 project. A total of 932 Spanish students participated in the current survey, the majority being women (see Table 1), and with all levels of university education represented. Most participants were students from one of the 3 public universities of the Autonomous Region of Galicia, an area in the Northwest of Spain. They were classified into 5 groups according to the branch of knowledge to which their undergraduate, master or Ph.D. program belonged: Arts and Humanities, Experimental Sciences, Health Sciences, Engineering and Architecture, and Social and Legal Sciences.

**TABLE 1:** Participants’ main demographic variables and psycho-social characteristics.

**Variables**	***N***	**%**
**Gender**		
Women	704	75.5
Men	222	23.8
Others	6	0.6
**Age**		
18–20	280	30.0
21–23	417	44.7
24–26	112	12.0
27–29	47	5.0
30 or more	76	8.2
**University**		
Universidade de Santiago de Compostela	661	70.9
Universidade de A Coruña	38	4.1
Universidade de Vigo	155	16.6
Others	78	8.4
**Level of education**		
Undergraduates	810	86.9
Postgraduates	85	9.1
Ph.D. students	37	4.0
**Branch of knowledge**		
Arts and humanities	161	17.3
Experimental sciences	111	11.9
Health sciences	391	42.0
Engineering and architecture	84	9.0
Social and legal sciences	183	19.7
**Self-perceived social class**		
Low	40	4.3
Low-middle	245	26.3
Middle	543	58.3
Middle-high	102	10.9
High	2	0.2
**Diagnosed with a mental health problem before quarantine**		
Yes	130	15.7
**During quarantine**		
Returned to parent’s home	504	56.0
**Being high risk COVID-19 people**		
Because of age	16	1.8
Due to previous diseases	102	11.3
Because being essential workers	20	2.2
No	770	85.6
**Having lived with other high risk COVID-19 people**		
Because of age	235	26.1
Due to previous diseases	307	34.1
Because they work in the medical field	109	12.1
Because being essential workers	317	35.2
No	290	32.2
**Suffered COVID-19**		
Myself	8	0.9
Someone close to me	206	22.9
No	704	78.2

When the demographics of the sample are compared to the overall population in the main university of Galicia (USC) and in the whole Spanish university system (SUE; see Table 2), we find that our sample is composed of a relatively higher proportion of women: 75.5% versus 61.1% and 55.3.% in USC and SUE, respectively. With respect to the branches of knowledge, most of the participants in our study were enrolled in programs related to Health Sciences (42.0%), Social and Legal Sciences (19.7%), and Arts and Humanities (17.3%); these proportions are very similar in the USC system (see Table 2), but not totally in the SUE system, where a higher proportion of undergraduates enroll in Social and Legal Science programs (47.1%). With regards to educational levels, the high representation of undergraduate students in our study (86.9%) was very similar to the proportion in both the USC (81.6%) and SUE (80.2%).

**TABLE 2:** Percentage of students enrolled in university studies as a function of gender, level of education and branch of knowledge in our study and for the USC and SUE systems.

	**% of students**		
**Variables**	**Our study**	**USC**	**Statistics for all Spanish universities (SUE)**
**Gender**			
Women	75.5	61.6	55.3
Men	23.8	38.4	44.7
Others	0.6	–	–
**Level of education**			
Undergraduates	86.9	81.3	80.2
Postgraduates	9.1	7.9	14.3
PhD students	4.0	10.8	5.5
**Branch of knowledge**			
Arts and humanities	17.3	13.2	10.4
Experimental sciences	11.9	11.9	6.5
Health sciences	42.0	30.5	18.3
Engineering and architecture	9.0	7.0	17.7
Social and legal sciences	19.7	37.4	47.1

*Data retrieved from the online resource “EDUCAbase”, created by the Spanish government. The statistics refer to the 2019–2020 academic year.*

A large percentage of the students (56.0%) in the sample moved back to their parents’ home during the pandemic and reported having lived with high risk COVID-19 people (83.4%) or being part of the high-risk population themselves (15.1%). While most of the participants had not suffered the COVID-19, 22.8% had lived in an environment with an infected person close by.

### Variables and Instruments

For the purposes of the present study, measures of psychological health, psychosocial stressors, and coping strategies were administered in the context of the broader COnVIDa-20 project.

#### Psychological Health

Four measures were used as indicators of psychological health. Specifically, instruments for the assessment of anxiety, depression, irritability, and self-perceived change in psychological health were administered.

##### Anxiety

Students were asked to respond to the 7-item Generalized Anxiety Disorder Scale (GAD-7; Spitzer et al., 2006). On the GAD-7 scale, symptoms of anxiety over the last 15 days (e.g., “Feeling nervous, anxious, or on edge”) were reported using a 4-point Likert rating scale ranging from 0 (not at all) to 3 (almost every day), and thus total scores ranged from 0 to 21. Scores of 5, 10, and 15 were taken as the cut-off points for mild, moderate, and severe anxiety, respectively. When used as a screening tool, scores of 10 or higher were taken as suggestive of a significant pattern of anxiety (García-Campayo et al., 2010). Cronbach’s α in this study was 0.89 (MIC = 0.54).

##### Depression

The Patient Health Questionnaire (PHQ-9; Kroenke et al., 2001) was administered as a measure of depression. Taking a 15-day period as a reference, participants were asked to rate the presence of depressive symptoms (e.g., “Feeling down, depressed, or hopeless”) using a 4-point Likert scale, from 0 (not at all) to 3 (nearly every day), and thus total scores ranged from 0 to 27 (Cronbach’s α = 0.87, MIC = 0.44). Scores of 5, 10, 15, and 20 were considered as cut-off points for mild, moderate, moderately severe, and severe symptoms, with scores of 10 or higher being indicative of possible depression.

##### Irritability

Since feelings of irritability have also been described as common effects of the isolation measures in health-related crises (Brooks et al., 2020), the Brief Irritability Test (BIT; Holtzman et al., 2015) was administered. This scale is composed of 5 items (e.g., “I have been feeling like I might snap”) with a 6-point Likert scale from 1 (never) to 6 (always). Cronbach’s α was 0.92, MIC = 0.71.

##### Self-perceived change in mental health

In order to measure subjective changes associated with the COVID-19 situation specifically, we asked students whether they had perceived changes in their mental health during quarantine, using a Likert scale ranging from 1 (my mental health is much worse) to 5 (my mental health is much better); therefore, a high score in this scale indicates that the participants self-perceived an improvement, whereas a low score indicates that the participants perceived a deterioration in their mental health.

#### Stressors Associated With the COVID-19 Context

We developed 26 items to assess possible sources of stress during the quarantine. Participants had to indicate, on a Likert scale, the extent to which they had been disturbed by these during the quarantine, with 1 being “not at all” and 4 “a lot”. These items were subjected to Principal Components Analysis with Varimax Rotation. Both scree test and Kaiser’s criteria recommended a meaningful 5-factor solution, which grouped the stressors in the following domains: Academic stressors (e.g., “Not receiving the academic training that one expected”), Social distancing (e.g., “Being required to stay at home for so long”), Pandemic (e.g., “The risk that either you or people close to you might become infected by COVID-19”), General overload (e.g., “Lack of free time”) and Interpersonal conflicts (e.g., “The intensification of family conflicts”). Scales were created for the five domains by averaging the items aligned with each factor, with Cronbach’s α ranging from 0.69 (Interpersonal conflicts) to 0.80 (Academic stressors), and MIC ranging from 0.34 (Social distancing) to 0.40 (Academic stressors). The distribution of items across scales is shown as Supplementary Table 1.

#### Coping Strategies in the COVID-19 Context

A set of 14 items was administered to assess the strategies displayed by participants in dealing with difficulties encountered in the COVID-19 situation. The items were mainly based on the Brief COPE questionnaire (Carver, 1997), and encompassed strategies potentially relevant to students in the quarantine: for instance, emotional support (“Looking for understanding and emotional support from others”), trying to actively improve the situation (“Concentrating my efforts on looking for a solution that might resolve the difficulties I’m facing”), instrumental support (“Trying to get help and advice from other people”), self-distraction (“Doing something to distract me from the difficulties I’m facing”), substance use (“Consuming alcohol or other substances to feel better”), spiritual attitude (“Trying to find solace in my spiritual or religious beliefs”), venting (“Expressing my negative feelings”), humor (“Trying to laugh at the situation”), and acceptance (“Accepting the reality of the fact that this is happening and adapting myself to the situation”). We also developed additional items specifically suited to the quarantine situation: routine maintenance (“Trying to maintain routines and schedules”), self-care (“Taking care of my health (nutrition, exercise…) to be psychologically stronger”) and helping others (“Helping others with their own difficulties”). The items were rated on a 4-point Likert scale, from 0 (not used) to 3 (used a lot). Principal Component Analysis with Varimax rotation led to the identification of four domains, based on scree and Kaiser’s rules. The items were grouped by the analysis (factor loadings of at least 0.40) as follows: Other-oriented coping, which includes both seeking and providing help (e.g., “Trying to get help and advice from other people”; “Helping others with their own difficulties”), Reframing (e.g., “Looking for something good in what is happening”; “Concentrating efforts on looking for a solution to resolve difficulties”), Disengagement Activities (e.g., “Doing something to distract me from the difficulties I’m facing”; “Doing relaxing activities”), and Structure/Healthy Routines (“Trying to maintain routines and schedules”; “Taking care of my health (nutrition, exercise…) to be psychologically stronger”). According to this distribution, items were averaged as a means of composing four coping scales, with Cronbach’s α ranging from 0.57 (Reframing) to 0.72 (Other-oriented), and MIC ranging from 0.31 (Reframing) to 0.40 (Other-oriented). The final scales are shown as Supplementary Table 2.

### Procedure

The questionnaires were conducted using an internal web application, which was available online from April 27th to May 27th. Students were invited to participate mainly via WhatsApp and were encouraged to spread the link to other students using the same platform, although other social media platforms like Twitter were also used. Hence a snowballing technique was used.

This study was approved by the Bioethics Committee of the Universidade de Santiago de Compostela, and prior to beginning the questionnaire, participants were provided with the aims and requirements of the study. They were also asked to give their explicit agreement to participate in the study and were informed that participation was completely anonymous and voluntary. On average, the survey took 20 min to complete and there was no reward or compensation for participating.

### Data Analysis

Firstly, descriptives for the stressors, coping domains, and indicators of psychological health were computed, with specific focus on percentages of participants scoring high in the measures that have cut-off points, i.e., anxiety and depression. Second, a multivariate path model was used to examine the relationships among sources of stress, coping strategies, and psychological health. Specifically, the significance of direct and indirect effects was tested through a path analysis in AMOS v24 using maximum likelihood bootstrapping techniques (5,000 bootstrap iterations) and bias-corrected 90% confidence intervals (Preacher and Hayes, 2008). Several fit indices were used to test the model fit, including the χ^2^ statistic, comparative fit index (CFI), and root mean square error of approximation (RMSEA). Models with a CFI value of 0.90 or higher (Hu and Bentler, 1999) and a RMSEA value below 0.08 (Browne and Cudeck, 1993) were considered to have an acceptable fit.

## Results

### Descriptives for the Studied Variables and Rates of Psychological Problems

Table 3 sets out main descriptives for the measures of psychological health, sources of stress, and coping strategies.

**TABLE 3:** Descriptives of the main variables in the study.

**Variables**	**Mean (SD)**	**Range**
**Psychological health**		
Anxiety	10.82 (5.00)	0–21
Depression	12.98 (6.46)	0–27
Irritability	16.88 (6.23)	5–30
Changes in mental health	2.19 (0.77)	1–5
**Stressors**		
Academic	3.06 (0.72)	1–4
Social distancing	2.94 (0.66)	1–4
Pandemic	2.92 (0.59)	1–4
General overload	2.84 (0.78)	1–4
Interpersonal conflict	1.99 (0.74)	1–4
**Coping**		
Other-oriented	1.48 (0.68)	0–3
Reframing	1.65 (0.63)	0–3
Structure/healthy routines	1.39 (0.84)	0–3
Disengagement activities	1.80 (0.75)	0–3

The descriptives for the scales of psychological health (anxiety, depression, and irritability) are relatively high for community populations, indicating a high average level of emotional disturbance. Regarding the analysis of stressors, this shows that academic stressors were rated as the most disturbing, on average, whereas interpersonal conflicts were the least disturbing. As for coping strategies, the involvement in disengagement activities (distracting and relaxing) was the most widely used by students during the quarantine, whereas the structure/healthy routines strategy was reported to have been the least used.

The descriptive statistics for each of the specific stressors and coping strategies are presented as Supplementary Tables 1 and 2. Among the stressors, the highest mean was achieved by “Uncertainty about the evaluation of the subjects you are taking” (mean = 3.45), followed by “The economic future of society as a consequence of the crisis” (mean = 3.40), “Uncertainty about the COVID-19 crisis” (mean = 3.36) and “Lack of face-to-face contact with loved ones” (mean = 3.30). Among the coping strategies, “Accepting the reality of the fact that this is happening and adapting myself to the situation” was the most used (mean = 2.02). Other strategies with high means were “Doing something to distract me from the difficulties I’m facing” (mean = 1.88), “Doing relaxing activities” (mean = 1.73), and “Helping others with their own difficulties” (mean = 1.72).

Regarding the rates of psychological problems, when the cut-offs for anxiety are taken into account, 61.2% of participants scored equal to or higher than 10, i.e., the cut-off usually considered for identifying significant anxiety, according to the norms of the scale; specifically, 38.8% showed moderate anxiety, and 22.4% severe anxiety. As for the depression scale, 65.8% of participants scored equal to or higher than 10, which is the usual cut-off taken as a reference for depression screening (Manea et al., 2012): 23.4% showed symptoms that were moderate, 25.2% ones that were moderately severe, and 17.2% severe symptoms.

When rates for anxiety and depression are compared across genders, significant differences are found. For anxiety, 63.8% of women and 52.8% of men scored above the cut-off (χ^2^ = 7.79, 1 df, *p* < 0.006). For depression, 68.0% of women and 58.8% of men surpassed the cut-off (χ^2^ = 5.68, 1 df, *p* < 0.02). The “others” gender could not be introduced into the comparisons due to the small size of the group.

Differences were also found for the level of university studies (χ^2^ = 12.02, 2 df, *p* < 0.002); the rates for anxiety were 63.5% (undergraduates), 45.1% (postgraduates), and 46.7% (Ph.D. students). For depression, the rates were 68.3, 47.9, and 50%, respectively (χ^2^ = 15.43, 2 df, *p* < 0.001). No differences were found across branches of academic knowledge.

The scores for self-perceived changes indicate that most participants felt that their mental health actually changed during the COVID-19 crisis, with a mean of 2.19 within a range from 1 (change to much worse) to 5 (change to much better). In terms of percentages, 14.7% perceived that they were much worse, 57.5% worse, 22.7% did not perceive any change, 4.2% perceived that they were better, with only 1% reporting that they felt much better.

Additional analysis by gender revealed that the mean change in women was worse than for men (2.15 vs. 2.32; *F*[1,822] = 7.25, *p* < 0.008). No differences were found across university levels or branches of academic knowledge.

### Multivariate Path Model

With the aim of analyzing how the different domains of stressors and coping strategies might have impacted psychological health, we then performed a path analysis. Gender and age were used as covariates in the model in order to control for effects on anxiety, depression, irritability, and self-perceived change. First, we tested a saturated model in which all the paths (both direct and indirect) were included. We then tested the fit of a reduced model, in which only the significant paths and covariances were retained.

Table 4 presents the correlations between the constructs used in the path analysis. All sources of stress, as well as coping strategies, correlated significantly with anxiety, depression, irritability, and self-perceived change, except for disengagement activities. The highest correlation was found between interpersonal conflict and irritability (*r* = 0.48), and the lowest between disengagement activities and depression (*r* = −0.09). As expected, anxiety, depression, and irritability correlated strongly with each other (*r*s ranged from 0.63 to 0.75) and were negatively correlated to self-perceived change (*r*s ranged from −0.57 to −0.50).

**TABLE 4:** Correlations between the constructs used in the path analysis.

**Variables**	**1**	**2**	**3**	**4**	**5**	**6**	**7**	**8**	**9**	**10**	**11**	**12**	**13**
(1) Academic		0.42***	0.45***	0.53***	0.39***	0.26***	−0.01	−0.09*	0.05	0.45***	0.42***	0.36***	−0.41***
(2) Social distancing			0.36***	0.45***	0.42***	0.34***	−0.05	0.00	0.11**	0.43***	0.40***	0.38***	−0.42***
(3) Pandemic				0.30***	0.29***	0.33***	0.09**	0.00*	0.12***	0.34***	0.24***	0.24***	−0.22***
(4) General overload					0.35***	0.26***	−0.02	−0.09*	−0.05	0.47***	0.43***	0.37***	−0.39***
(5) Interpersonal conflict						0.13***	−0.04	−0.04	−0.03	0.45***	0.43***	0.48***	−0.35***
(6) Other oriented							0.27***	0.23***	0.31***	0.26***	0.15***	0.17***	−0.18***
(7) Reframing								0.35***	0.35***	−0.15***	−0.21***	−0.13***	0.24***
(8) Structure/healthy routines									0.30***	−0.12***	−0.28***	−0.10**	0.20***
(9) Disengagement activities										−0.06	−0.09**	−0.03	0.09**
(10) Anxiety											0.75***	0.70***	−0.57***
(11) Depression												0.63***	−0.57***
(12) Irritability													−0.50***
(13) Self-perceived change													

*p < 0.05; **p < 0.01, ***p < 0.001; the correlations that remaining significant after Bonferroni’s correction (p < 0.0006) are displayed in bold characters.

**FIGURE 1:**
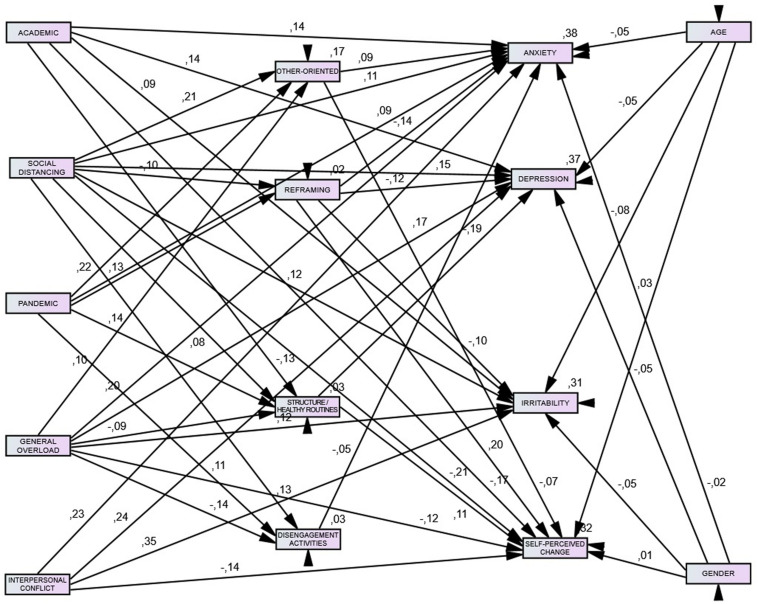
Relationship between stressors, coping, and health-related indicators. All reported path coefficients (*p* < 0.05) are standardized estimates. Terms of error, correlations, and covariances were omitted for clarity.

Figure 1 shows the final model in which only the significant paths and covariances were retained. The final model fits the data well, χ^2^ = 237.40, df = 37, *p* < 0.001, RMSEA = 0.08, CFI = 0.95, and predicted variation in health to an acceptable degree; explained variance ranged from 31% (irritability) to 38% (anxiety).

Standardized regression (β) weights were used to examine the size and significance of the direct effects of the stressors specified within the model (Byrne, 2016). Tables 5, 6 present coefficients from the path analysis model, after controlling for gender and age.

**TABLE 5:** Standardized and unstandardized regression weights, standard errors, *z*-values, and associated *p*-values for coping.

**Coping**		**Stressor**	**SRW**	**URW**	***SE***	**C.R.**	***p*-value**
Other oriented	←	Social distancing	0.20	0.20	0.03	5.58	0.000
Other oriented	←	Pandemic	0.22	0.24	0.03	6.35	0.000
Other oriented	←	General overload	0.10	0.09	0.03	3.01	0.003
Reframing	←	Social distancing	−0.10	−0.09	0.03	−2.77	0.005
Reframing	←	Pandemic	0.13	0.13	0.03	3.54	0.000
Structure/healthy routines	←	Academic	−0.13	−0.19	0.04	−3.32	0.000
Structure/healthy routines	←	Social distancing	0.08	0.10	0.05	2.06	0.039
Structure/healthy routines	←	Pandemic	0.13	0.19	0.05	3.48	0.009
Structure/healthy routines	←	General overload	−0.09	−0.09	0.04	−2.30	0.021
Disengagement activities	←	Social distancing	0.12	0.14	0.04	3.21	0.001
Disengagement activities	←	Pandemic	0.11	0.14	0.04	2.96	0.003
Disengagement activities	←	General overload	−0.14	−0.13	0.03	−3.87	0.000

*SRW, standardized regression weights; URW, unstandardized regression weights; SE, standard error.*

**TABLE 6:** Standardized and unstandardized regression weights, standard errors, *z*-values, and associated *p*-values for health-related indicators.

**Health-related indicator**		**Coping**	**SRW**	**URW**	***SE***	**C.R.**	***p*-value**
Anxiety	←	Other oriented	0.08	0.61	0.17	3.63	0.000
Anxiety	←	Reframing	−0.14	−1.09	0.22	−4.84	0.000
Anxiety	←	Disengagement activities	−0.05	−0.32	0.14	−2.26	0.023
Depression	←	Reframing	−0.12	−1.24	0.29	−4.28	0.000
Depression	←	Structure/Hhealthy routines	−0.18	−1.37	0.17	−7.96	0.000
Irritability	←	Reframing	−0.10	−0.97	0.28	−3.42	0.000
Self-perceived change	←	Other oriented	−0.07	−0.08	0.03	−2.43	0.015
Self-perceived change	←	Reframing	0.19	0.24	0.03	6.33	0.000
Self-perceived change	←	Structure/healthy routines	0.10	0.09	0.02	3.63	0.000

*SRW, standardized regression weights; URW, unstandardized regression weights; SE, standard error.*

Results of the path analysis revealed that pandemic stressors showed a significant positive association with all the coping strategies. Social distancing was positively associated with other-oriented strategies, routines, and disengagement activities, but negatively associated with reframing. In contrast, general overload had a significant negative association with routines and disengagement activities, and a positive one with other-oriented strategies. Academic stressors only had a direct and significant negative effect on routines.

Regarding the coping-psychological health pathway, reframing was associated with better psychological health consistently across measures, that is, less anxiety, less depression, less irritability, and fewer unfavorable changes in mental health. The negative association of routines with depression was particularly strong (β = −0.19, *p* < 0.001). Conversely, other-oriented coping predicted higher levels of psychological ill-being: more anxiety (β = 0.08, *p* < 0.001) and the perception of feeling worse, as indicated by the negative coefficient linking other-oriented and self-perceived health change (β = −0.07, *p* < 0.05). The coping domain involved in psychological outcomes to the least extent was that of disengagement activities, which had no significant effect.

Furthermore, once adjusted for confounding variables, decomposition of total effects (Table 7) showed that both the direct and indirect effects of sources of stress on psychological measures were statistically significant (*p* < 0.05), providing a definite pattern: Higher general overload, social distancing, interpersonal conflict, and academic stressors were significantly associated with higher anxiety, depression, and irritability, and self-perceived change to a worse mental health. Pandemic stressors, in turn, only had a direct effect on anxiety, β = 0.092, *p* < 0.001.

**TABLE 7:** Path analysis testing the indirect effects of the variables that entered into the model.

**Variables**	**Total effects β (90%CI)**	**Direct effect β (90%CI)**	**Indirect effect β (90%CI)**
**Anxiety**			
General overload	0.21***(0.15,0.27)	0.20***(0.14,0.26)	0.01**(0.00,0.02)
Pandemic	0.08**(0.04,0.12)	0.09***(0.05,0.13)	−0.00(−0.01,0.00)
Social distancing	0.13***(0.07,0.19)	0.11**(0.05,0.17)	0.02**(0.01,0.04)
Interpersonal conflict	0.23***(0.17,0.28)	0.23***(0.17,0.28)	–
Academic	0.14***(0.07,0.20)	0.14***(0.07,0.20)	–
**Depression**			
General overload	0.19***(0.13,0.25)	0.17***(0.11,0.23)	0.01*(0.00,0.03)
Pandemic	−0.04***(−0.06,−0.02)	–	−0.04***(−0.06,−0.02)
Social distancing	0.15***(0.08,0.21)	0.15***(0.09,0.21)	−0.00(−0.02,0.01)
Interpersonal conflict	0.23**(0.18,0.29)	0.23**(0.18,0.29)	–
Academic	0.16***(0.10,0.22)	0.13***(0.08,0.19)	0.02**(0.01,0.03)
**Irritability**			
General overload	0.12**(0.06,0.18)	0.12**(0.06,0.18)	–
Pandemic	−0.01***(−0.02,−0.00)	–	−0.01***(−0.02,−0.00)
Social distancing	0.12***(0.06,0.18)	0.11**(0.05,0.17)	0.01**(0.00,0.02)
Interpersonal conflict	0.35***(0.29,0.40)	0.35***(0.29,0.40)	–
Academic	0.09*(0.03,0.15)	0.09*(0.03,0.15)	–
**Self-perceived change**			
General overload	−0.14**(−0.20,−0.07)	−0.12**(−0.18,−0.05)	−0.01**(−0.03,−0.00)
Pandemic	0.02*(0.00,0.04)	–	0.02*(0.00,0.04)
Social distancing	−0.23***(−0.30,−0.17)	−0.20***(−0.27,−0.14)	−0.02*(−0.04,−0.00)
Interpersonal conflict	−0.13**(−0.19,−0.07)	−0.13**(−0.19,−0.07)	–
Academic	−0.18***(−0.25,−0.17)	−0.16***(−0.23,−0.10)	−0.01**(−0.02,−0.00)

* p < 0.05, ** p < 0.01, *** p < 0.001.

The final model revealed 13 indirect pathways among sources of stress and psychological health measures throughout the model; however, the proportion of mediated effects (%) were weaker in magnitude, ranging from 7.34 to 18.84%. The indirect effects of social distancing (β = −0.002, 90% CI: −0.021, 0.016, *p* > 0.10) and the pandemic (β = −0.005, 90% CI: −0.019, 0.009, *p* > 0.10) on depression and anxiety, respectively, were no longer significant.

## Discussion

A growing number of studies have addressed the psychological effects of the COVID-19 crisis, but very little is known about the impact on university students, even if students might be expected to be greatly affected by the pandemic conditions and by the policies implemented to curtail the spread of the disease. Emergent adulthood is itself a developmental time and is well-known to be vulnerable to psychological difficulties (Schulenberg et al., 2004); in particular, high rates of mental problems have previously been reported in student populations (Auerbach et al., 2018). The COVID-19 crisis led to the closure of universities and forced students not only to change their general life conditions, but also to substantially adjust their daily academic work, long-term projects, and their expectations. Hence, the current study examined the psychological health of university students during the Spanish quarantine, considering the specific sources of stress perceived by these students during the crisis, as well as the strategies reported to have been used to cope with the situation of COVID-19.

### Mental Health During the COVID-19 Crisis

Several indicators of mental health were analyzed in this study. On the one hand, we included measures of common psychological problems (anxiety and depression), using standardized measures widely employed in previous research for prevalence purposes (Quon et al., 2015), and also in recent research looking at the COVID-19 crisis (Zhu et al., 2020). On the other hand, we also included a measure of irritability (proneness to anger, annoyance, frustration, and aggressive reactions), which previous studies have identified as a possible outcome of social distancing measures (Brooks et al., 2020), especially in young people (Balluerka et al., 2020). Additionally, as a specific indicator of how well-being evolved in relation to the COVID-19 crisis, we asked students about self-perceived changes in their mental health. Across this array of measures, our results consistently suggest that students experienced considerable psychological difficulties during the acute part of the crisis. The numbers for anxiety and depression are very high, with 61.2% of participants scoring above the GAD-7 usual cut-off, and 65.8% surpassing the PHQ-9 cut-off. High levels of irritability were also found, and more than 70% of students reported that their mental health had worsened during the confinement. When the means of the scales are considered, they are also high compared to those reported in previous studies on community populations (Löwe et al., 2008; García-Campayo et al., 2010; Holtzman et al., 2015).

The finding that students show high rates of psychological problems is consistent with studies conducted on the general population, which have found that young people are psychologically more affected by the COVID-19 crisis than older people (Valiente et al., 2020). Those few previous studies that have specifically examined psychological health in university students during the pandemic also tend to report high levels of anxiety, depression, stress (Cao et al., 2020; Odriozola-González et al., 2020) and even suicidality (Patsali et al., 2020). Although, due to the diversity of measures employed, the levels of psychological disturbance previously reported in students’ samples are difficult of compare, the rates found in our study seem to be strikingly high. This may be due to the critical time when our data was collected, that is, after more than 6 weeks of home confinement, and also, perhaps more importantly for the student population, close to the end of the academic year, with final exams to be taken in uncertain and unprecedented conditions.

In accordance with past research on general psychological health (Salk et al., 2017), and with some other studies conducted during the COVID-19 crisis (Wang et al., 2021), higher rates of emotional difficulties were found for women than for men. In the absence of longitudinal data, we cannot disentangle the issue of how far these differences reflect the higher prevalence of common psychological problems in women, and/or a higher impact of the crisis on women. Nevertheless, we found gender differences not only for mental health measures (current anxiety, depression, and irritability), but also for the measure of self-perceived *change*; i.e., women perceived that their mental health deteriorated more, and this might suggest that women were more affected than men by the COVID-19 crisis. Along these lines, it has been suggested that a higher perception of threat and a greater sensitivity to the loss of control may influence the higher vulnerability of women in situations of crisis and trauma (Olff et al., 2007). And, in relation to COVID-19, it has also been suggested that the pandemic may differentially affect women by the worsening of gendered burdens, such as the overload derived from household or caregiving tasks (McLaren et al., 2020).

Our results also indicate that more psychological difficulties are found in undergraduate than in postgraduate students. Apart from age-related differences, which may involve less resilience in the adaptation to drastic changes (Masten et al., 2006), undergraduate students might have been more greatly affected by modifications in teaching and evaluation, as undergraduate learning is usually less autonomous and more guided by lectures and other on-site activities that were banned during the confinement. Moreover, studies conducted in pre-pandemic times have reported more psychological problems in undergraduates than in graduate students (Wyatt and Oswalt, 2013), so differences in pre-existing mental health cannot be precluded. In any case, this pattern of results suggests the need to promote mental health in the undergraduate population, in that they stand out as the student group most susceptible to emotional difficulties, in both ordinary and crisis periods.

### Stress and Coping During the COVID-19 Crisis

Although this study could not ensure the representativity of the sample, overall, our results suggest that psychological distress was high among university students during the Spanish quarantine. With the aim of making advances in the identification of determinants that may help to explain these disturbances, we explored the sources of stress as perceived by students during the pandemic situation. Our results showed that psychological difficulties were related to the experience of several domains of stressors, such as academic future, task overload, worsening of interpersonal conflicts, and restrictions in pleasant social contact. So, it seems that the personally relevant stressors, linked to difficult and troubling academic and social experiences, were the ones that were most involved in students’ psychological problems. These results are in line with pre-pandemic reports on the main stressors for university students; for example, Beiter et al. (2015) found that academic performance, pressure to succeed, and relations with friends were among the top concerns for a sample of American unviersity students. The findings of the other studies conducted during the pandemic have also pointed to academic and relational worries as the main determinants of alterations in the mental health of students (Elmer et al., 2020).

According to our results, stressors related to the spread of the disease (lethality, risk of contagion) were less associated with psychological distress; students seemed to be less vulnerable to health and society-wide concerns which, although relevant, might be perceived to be more distant and to have fewer personal implications. The message that young people were less affected by the disease (Liao et al., 2020) was quickly disseminated from the very first weeks of the pandemic; also, low risk appraisals and a sense of invulnerability have previously been described as features of adolescents and young adults (cf. Millstein and Halpern-Felsher, 2002; Lapsley and Hill, 2010). Age-related processes and widespread media messages, then, might both have affected the psychological resistance of students in relation to the threats of the disease. These same processes might help explain the high number of contagions in young Spanish people once the social restrictions were reduced (Minder, 2020).

This study also examined coping strategies, i.e., the efforts made by students to deal with the stressful conditions arising from the COVID-19 crisis. We used a measure specifically aimed at capturing context-relevant coping, which allowed us to inductively identify four ways of coping during the acute phase of the crisis: focusing on others to ask for support or to offer help (Other-oriented), accepting the uncontrollable nature of the crisis, focusing on positive dimensions, and trying to take steps to change what is controllable (Reframing), getting involved in activities which can help one to detach oneself from stressful situations (Disengagement activities), and keeping/setting up a healthy structure in one’s daily life (Structure/Healthy routines). It is remarkable that the first three dimensions, based closely on the Brief COPE (Carver, 1997), and less specifically related to COVID-19 context, tightly resemble other coping solutions found in emerging adulthood (e.g., Jenzer et al., 2019), which may indicate that they can be seen as robust coping domains for this life stage.

When we analyze the relationships between coping and psychological health, we find that, despite being one of the most used types of coping in our sample, disengagement activities are almost unrelated to psychological adjustment. In the literature on coping, some controversies remain as to the efficacy of disengagement coping to deal with different kinds of life stressors (Waugh et al., 2020); in the specific context of the pandemic, our results suggest that devoting time to distracting/relaxing activities might have some minimal effect on the psychological health.

We also found that another of the most used coping strategies in our study, other-oriented coping, is associated with higher levels of psychological disturbances. Our “other-oriented” scale, as empirically delimited by factor analysis, joins together both asking and giving help, thus defining an affiliative coping style which turns to other persons with the aim of reducing stress. Our results on the negative effects of this style are rather unexpected, since social support is usually considered a protecting mechanism in stressful situations (Ozbay et al., 2007), including the COVID-19 pandemic (Cao et al., 2020), and in demanding academic conditions (Rayle and Chung, 2007). Likewise, helping behaviors have been assumed to be a means of coping with collective crises (e.g., Balluerka et al., 2020), as they can be a source of fostering good moods, a sense of self-efficacy, and a way of promoting social integration (Vollhardt, 2009). Nevertheless, our finding that other-oriented coping does not enhance well-being is not an isolated result within research into coping, particularly with young samples (Braun-Lewensohn et al., 2010). It has been tentatively suggested that reliance on others could sometimes be an ultimate resource for severely troubled people who have previously tried other ways of dealing with difficult situations (Okafor et al., 2016). Additionally, it has been proposed that the emphasis on reaching out to others might be an index of personal dependence (Lewis and Frydenberg, 2002) and might hamper the development of more self-reliant coping resources. Although more research on these lines is needed, it is even possible that in a large-scale crisis like COVID-19, where social connectedness is hindered, *seeking* social support needs to be clearly distinguished from *obtaining* satisfactory social support; with all the population affected at the same time by the same risks, and with social distancing in force, trying to help or be helped by others may not have been as effective as might have been in more common stress situations, where just one or a few individuals are directly affected (e.g., personal illness and interpersonal breakup). In this line, recent research showed that altruistic students, willing to help others, suffered more emotional difficulties during the COVID-19 pandemic, as the external difficulties to behave pro-socially could bring them a sense of low self-efficacy (Feng et al., 2020).

Other coping strategies were found to be associated with low levels of mental health problems. One of these is to accept and cognitively re-appraise the situation (reframing), attempting to seize on positive aspects and to solve the problems that remain controllable. In previous research, that coping strategy, which is usually considered as part of the so-called “active coping” (e.g., Gaudreau, 2017), has shown its capacity to predict mental health and achievement outcomes in a number of psychopathological areas, and it is usually self-perceived as effective by the persons who display it (Crocker et al., 2015); in addition, interventions aimed to boost active coping have proved to be successful for stress management (Jamieson et al., 2018). Based on our results, the promotion of reframing skills could be recommended as a potentially useful way to develop resilient attitudes among university students.

During the acute phases of the pandemic, health agencies and the mass media have recommended setting up regular schedules and routines in daily life (e.g., El Camino Health, 2020; World Health Organization [WHO], 2020b) in terms of work, eating, leisure time, exercising and sleeping, in that these might bring some regularity in the midst of uncertainty, and might prevent perturbations in mood and psychobiological rhythms. Our results seem to endorse such recommendations: students who kept regular schedules and/or established healthy routines as a way of coping showed better outcomes in mental health. Our results also reinforce the specific connection between routines and mood/depression problems, which was highlighted in previous research (Boland et al., 2019). Given that depression is one of the most prevalent disorders in university students, both before and during the pandemic (Auerbach et al., 2018; Odriozola-González et al., 2020), this result might guide preventive interventions to help students remain healthy and to cope with crises such as the COVID-19 confinement.

In general, this study shows that what students do to cope with the situation is relevant to an understanding of individual differences in mental health during the time of COVID-19. In fact, as evidenced by our structural model, the effect of stressors on mental health is conveyed, in part, through students’ coping efforts. In other words, coping strategies emerge as proactive actions that may substantially affect the experience of the crisis, thus opening roads for psychological inoculation amidst the COVID-19 pandemic.

### Limitations and Future Research

This study is not exempt from limitations. First, the sample was based on a snowballing technique, which cannot guarantee representativeness in terms of the Spanish population of university students. For example, a big proportion of our sample (two-thirds) was composed of women. As we indicated in the Methods section, more women than men are enrolled in the Spanish university system, and, even more in the USC, i.e., the university that was the major source of participants for our study. Nevertheless, the number of women in our sample is still disproportionate. This gender asymmetry is commonly found in studies with similar aims to ours (e.g., Husky et al., 2020; Patsali et al., 2020), and it could indicate that women are more willing to participate in this type of projects. It is also worth noting that the questionnaire seems to have been more disseminated among students in educational programs of health, social sciences, and arts, where the predominance of women is particularly high. Thus, although the sample was of a considerable size and variety in terms of academic fields, educational levels, and socioeconomic origins, sampling bias recommends some caution, especially in relation to general prevalences and descriptive results.

Second, as with most studies in this area, self-reports were the only measurement technique, raising the possibility that shared method variance inflated the associations among variables to some extent. Thirdly, as noted above, this is a cross-sectional study that could not consider data on participant’s previous anxiety, depression, or any other relevant clinical diagnosis from before COVID-19, and therefore could not identify and eliminate the proportion of psychological problems already present before the pandemic. The lack of longitudinal data also makes it impossible to accurately identify the directionality of the effects; even when the flow from stressors/coping to mental health is theoretically driven (Carver and Connor-Smith, 2010), and is coherent with a vast number of empirical reports (Cooper and Quick, 2017), reciprocal effects cannot be discarded. Perception of stress may be influenced by psychological disturbances, and even more than this, stressful events might be precipitated by psychological problems, according to stress-generation models (Rudolph et al., 2000). For instance, depressed or irritable individuals may worsen interpersonal conflicts during the pandemic due to their inappropriate, unstable, or offensive behavior.

Thus, further research should address the bidirectional dynamics between stressors, coping, and mental health. Longitudinal designs will also allow for the delineation of the stability and change of the psychological disturbances as the COVID-19 conditions evolve, in order to ascertain to what extent those psychological difficulties depicted during the first phases of the pandemic were acute peaks or sustained reactions.

For now, this study is one the first to concurrently examine stressors, coping strategies, and mental health in university students during a critical point of the pandemic. We considered stressors that may be shared by the general population along with student-specific ones; we also measured multiple relevant coping behaviors, and we analyzed a variety of mental health measures. Our results provide a nuanced picture of how students were psychologically damaged during the first weeks of the crisis, when difficulties were the most impactful, and how they tried to face the challenges brought about by COVID-19.

### Implications

Our results have practical implications for interventions in university settings. By delineating the sources of stress and coping behaviors, we may be in a better position to boost endurance during the next phases of the pandemic as well as in any future crisis. Mental problems in university students are not only a matter of community health; psychological disturbances have an influence on academic performance, student retention, graduation rates and career development (Wyatt and Oswalt, 2013), and the university context is a privileged setting to promote mental health in emerging adulthood, as educational programs and university health centers can efficiently reach a wide number of emotionally vulnerable young adults. Our results suggest that monitoring mental health in universities may lead to the identification of many students who are susceptible to benefit from assistance in social/health crises. Counseling services, delivered in online formats (Zhai and Du, 2020), may be a cost-efficient way of reaching vulnerable students; online interventions show the added advantages of addressing other barriers to treatment such as stigma and inconvenience, as pointed out elsewhere (Auerbach et al., 2018). While universities are unlikely to have enough resources for the treatment of severe cases, they might be able to offer screening services, along with first-aid interventions, which may refer students to specialized services when needed.

In terms of specific interventions, our results suggest the appropriateness of training in coping skills for acceptance, reframing, and healthy structuring of one’s daily life, even when the future is uncertain, and when external schedules are lacking. Different psychological orientations may provide fruitful insights for such interventions, including acceptance and commitment therapy, cognitive-behavioral therapy, and behavioral activation approaches (Polizzi et al., 2020). While students are an asset for universities and more broadly for society, their mental health has been shown to have a certain fragility; the need for prevention and health promotion emerges as a general take-home message from the current evidence on COVID-19 outcomes.

## Data Availability Statement

The raw data supporting the conclusions of this article will be made available by the authors, without undue reservation, to any qualified researcher.

## Ethics Statement

The studies involving human participants were reviewed and approved by Bioethics Committee of the Universidade de Santiago de Compostela. The patients/participants provided their written informed consent to participate in this study.

## Author Contributions

IF and ER were involved in the conceptualization of the project and the design of the study. IF, LV, CM, and ER elaborated the questionnaire. All authors were involved in the acquisition of the data. IP, CM, and ER wrote sections of the manuscript. CM and ER performed the statistical analysis. IF, CM, and ER were involved in the interpretation of the data. All authors were involved in the drafting and revising of the work for intellectual content, agreed to be accountable for the accuracy and integrity of the project, and approved the final version of the manuscript.

## Conflict of Interest

The authors declare that the research was conducted in the absence of any commercial or financial relationships that could be construed as a potential conflict of interest.
