# Supplementary material for: A Study on the Psychological Wound of COVID-19 in University Students
Source: Front Psychol. 2021 Jan 26;12:589927. doi: 10.3389/fpsyg.2021.589927 (PMC7870473; doi:10.3389/fpsyg.2021.589927)
Supplement: Supplementary file 1 [file Table_1.PDF]

Supplementary Material

Table 1S. Distribution of items across the five stressor domains, along with means and SDs for this study.

Stressors	Mean (SD)	Range
**Academic stressors (α = 0.80, MIC = 0.40)**		
Academic future in the long-term	3.16 (0.98)	1-4
Academic record damages due to the COVID-19 crisis	2.91 (1.08)	1-4
Laboral future	3.11 (1.06)	1-4
Not receiving the expected academic training	3.16 (0.95)	1-4
Uncertainty about academic evaluation	3.45 (0.91)	1-4
Financial difficulties	2.60 (1.07)	1-4
**Social distancing (α = 0.71, MIC = 0.34)**		
Lack of face-to-face contact (with loved ones)	3.30 (0.82)	1-4
Staying at home for so long	2.98 (1.00)	1-4
Upcoming lifestyle changes due the COVID-19 crisis	3.12 (0.89)	1-4
Loneliness	2.61 (1.13)	1-4
Delaying or cancelling plans	2.73 (1.00)	1-4
**Pandemic (α = 0.73, MIC = 0.36)**		
The risk of loved ones (or yourself) become infected	2.90 (0.90)	1-4
The human losses (caused by COVID-19)	3.06 (0.88)	1-4
Someone close (or yourself) being affected by COVID-19	1.93 (1.01)	1-4
The economic future of society	3.40 (0.71)	1-4
The uncertainty about the COVID-19 crisis	3.36 (0.76)	1-4
**General overload (α = .71, MIC = 0.39)**		
The academic homework overload	3.12 (1.06)	1-4
Difficulties adapting to online learning	2.93 (1.06)	1-4
Lack of free time	2.53 (1.12)	1-4
The impossibility of dedicating time to hobbies and interests	2.80 (1.00)	1-4
**Interpersonal conflicts (α = 0.69, MIC = 0.35)**		
(Intensification of) family conflicts	2.26 (1.12)	1-4
Worsening cohabit relations	2.50 (1.08)	1-4
Looking after other people	1.68 (0.91)	1-4
Breaking-up with partner	1.53 (0.96)	1-4

Table 2S. Distribution of items across the four domains of coping strategies, along with means and SDs for this study.

Coping Strategies	Mean (SD)	Range
**Oriented to others (α = 0.72, MIC = 0.40)**		
Looking for understanding and emotional support from others	1.63 (0.95)	0-3
Trying to get help and advice from other people	1.15 (0.88)	0-3
Expressing negative feelings	1.44 (0.99)	0-3
Helping others with their own difficulties	1.72 (0.81)	0-3
**Reframing (α = 0.57, MIC = 0.31)**		
Accepting the fact that this is happening and adapting to the situation	2.02 (0.77)	0-3
Looking for something good in what is happening	1.35 (0.96)	0-3
Concentrating efforts on looking for a solution to resolve difficulties	1.57 (0.81)	0-3
**Activities (α = 0.70, MIC = 0.54)**		
Doing something to be distracted from difficulties	1.88 (0.87)	0-3
Doing relaxing activities	1.73 (0.85)	0-3
**Structure/Healthy routines (α = 0.63, MIC = 0.46)**		
Trying to keep some routines and schedules	1.45 (0.97)	0-3
Taking care of one's health (nutrition, exercise…) to be psychologically stronger	1.35 (1.01)	0-3
**Items which did not load in any factor**		
Trying to find relief in my spiritual or religious beliefs	0.25 (0.61)	0-3
Making fun of the situation	1.23 (0.97)	0-3
Consuming alcohol or other substances to feel better	0.29 (0.67)	0-3
